# Critical Role of the Fusion Protein Cytoplasmic Tail Sequence in Parainfluenza Virus Assembly

**DOI:** 10.1371/journal.pone.0061281

**Published:** 2013-04-12

**Authors:** Raychel Stone, Toru Takimoto

**Affiliations:** Department of Microbiology and Immunology, University of Rochester School of Medicine and Dentistry, Rochester, New York, United States of America; Faculty of Biochemistry Biophysics and Biotechnology, Jagiellonian University, Poland

## Abstract

Interactions between viral glycoproteins, matrix protein and nucleocapsid sustain assembly of parainfluenza viruses at the plasma membrane. Although the protein interactions required for virion formation are considered to be highly specific, virions lacking envelope glycoprotein(s) can be produced, thus the molecular interactions driving viral assembly and production are still unclear. Sendai virus (SeV) and human parainfluenza virus type 1 (hPIV1) are highly similar in structure, however, the cytoplasmic tail sequences of the envelope glycoproteins (HN and F) are relatively less conserved. To unveil the specific role of the envelope glycoproteins in viral assembly, we created chimeric SeVs whose HN (rSeVhHN) or HN and F (rSeVh(HN+F)) were replaced with those of hPIV1. rSeVhHN grew as efficiently as wt SeV or hPIV1, suggesting that the sequence difference in HN does not have a significant impact on SeV replication and virion production. In sharp contrast, the growth of rSeVh(HN+F) was significantly impaired compared to rSeVhHN. rSeVh(HN+Fstail) which expresses a chimeric hPIV1 F with the SeV cytoplasmic tail sequence grew similar to wt SeV or rSeVhHN. Further analysis indicated that the F cytoplasmic tail plays a critical role in cell surface expression/accumulation of HN and F, as well as NP and M association at the plasma membrane. Trafficking of nucelocapsids in infected cells was not significantly affected by the origin of F, suggesting that F cytoplasmic tail is not involved in intracellular movement. These results demonstrate the role of the F cytoplasmic tail in accumulation of structural components at the plasma membrane assembly sites.

## Introduction

Sendai virus (SeV), the prototypical parainfluenza virus is composed of six major structural proteins: hemagglutinin-neuraminidase (HN), fusion (F), matrix (M), nucleocapsid (NP), phospho (P) and large (L) proteins. The two surface glycoproteins, HN and F, are responsible for attachment and fusion, and the M protein acts as a scaffold that bridges interactions between the viral envelope proteins and viral nucleocapsid (vRNP) that is composed of genomic RNA encapsidated with NP and associated with the polymerase P-L complex [1], [2]. The assembly process of parainfluenza virus involves multiple viral components with coordinated localizations. These components include the viral glycoproteins, which are transported to the plasma membrane through the exocytic pathway [3], [4], [5], and other viral proteins, such as the vRNPs, which utilize the recycling endosome pathway to reach the cell surface [6]. Role of recycling endosomes in virus assembly has also been suggested in some negative strand RNA viruses, such as respiratory syncytial virus (RSV) and influenza A virus [7]. M proteins are likely to be transported to the plasma membrane in part by an association with envelope glycoproteins [8]. In virions, the M protein is found underneath the envelope and interacts with both envelope glycoproteins and vRNPs [9]. This would suggest that the M protein acts as an organizer of viral components to concentrate the proteins at a defined budding site at the plasma membrane [3]. M protein binds lipid membranes both *in vitro* and *in vivo* and when purified singly the M protein self-assembles into ordered structures [10], [11]. A recent study using cryoelectron tomography showed that M dimers assemble into psudotetrameric arrays in the virions [9]. Co-expression of M and NP results in the production of virus-like particles (VLP) containing vRNP-like structures [12]. In SeV, with temperature sensitive M protein, the absence of M protein at non-permissive temperatures inhibits viral assembly [13], [14], [15]. Other studies with cells persistently infected with SeV, which expressed an unstable M protein showed a correlation with reduced virion formation [16].

The role of envelope glycoproteins in virus assembly is less clear, although a specific interaction between the glycoprotein cytoplasmic tails and the M protein of parainfluenza viruses has been thought to be important in the assembly and budding processes. In the case of parainfluenza virus 5 (PIV5), M and glycoproteins co-localize unless the cytoplasmic tail of HN or the cytoplasmic tails of both HN and F have been truncated [17], [18], [19]. SeV M becomes raft-associated only when co-expressed with the glycoproteins, which intrinsically sort to raft membranes [4]. These results suggest that M and glycoproteins assemble at specific locations on plasma membranes through interactions between M and the glycoprotein cytoplasmic tails. However, the contribution of HN and F in virus budding and virion formation is likely to differ between viruses. Previous studies showed that SeV HN was superfluous for virion budding [20], [21], [22], [23], [24]. In contrast, lack of SeV F reduced production of virions from infected cells, highlighting differences in the role of glycoproteins in virion formation and release [24]. Experiments involving use of recombinant SeV (rSeV) generated to express F and HN glycoproteins containing various truncations or amino acid substitutions in the cytoplasmic tail domains showed that loss of the cytoplasmic tail of F protein severely reduced virion production [25]. Other paramyxoviruses have also been shown to require the F protein cytoplasmic tail. Deletion of the cytoplasmic tail of the RSV F protein resulted in a failure to assemble RSV proteins into virus-like filaments, causing a reduction of viral titers by up to 1,000 fold [26], [27]. Similarly, mumps virus also requires the F protein cytoplasmic tail [28], and the cytoplamic tail of F with full length G protein was shown to be required for assembly in Hendra virus infections [29]. However, studies of PIV5 suggest a marked defect in virus budding and release when the HN cytoplasmic tail was deleted, while F cytoplasmic tail was dispensable for normal viral budding [19]. These studies highlight the role of glycoprotein cytoplasmic tails in virion production, but it is unclear how the HN or F cytoplasmic tails individually contribute to virus assembly and production and the contribution differs among paramxyoviruses.

In this study, we determined the role and specificity of the glycoprotein cytoplasmic tail sequences in virus assembly using closely related SeV and human parainfluenza virus type 1 (hPIV1). We rescued and characterized various recombinant SeVs containing hPIV1 HN or both HN and F. Our data indicate the critical role of the F cytoplasmic tail in accumulation of viral envelope proteins and vRNP at the plasma membrane, which is essential for efficient virion assembly and release.

## Results

### Rescue and characterization of rSeVhHN, rSeVh(HN+F) and rSeVh(HN+Fstail)

**Figure 1:**
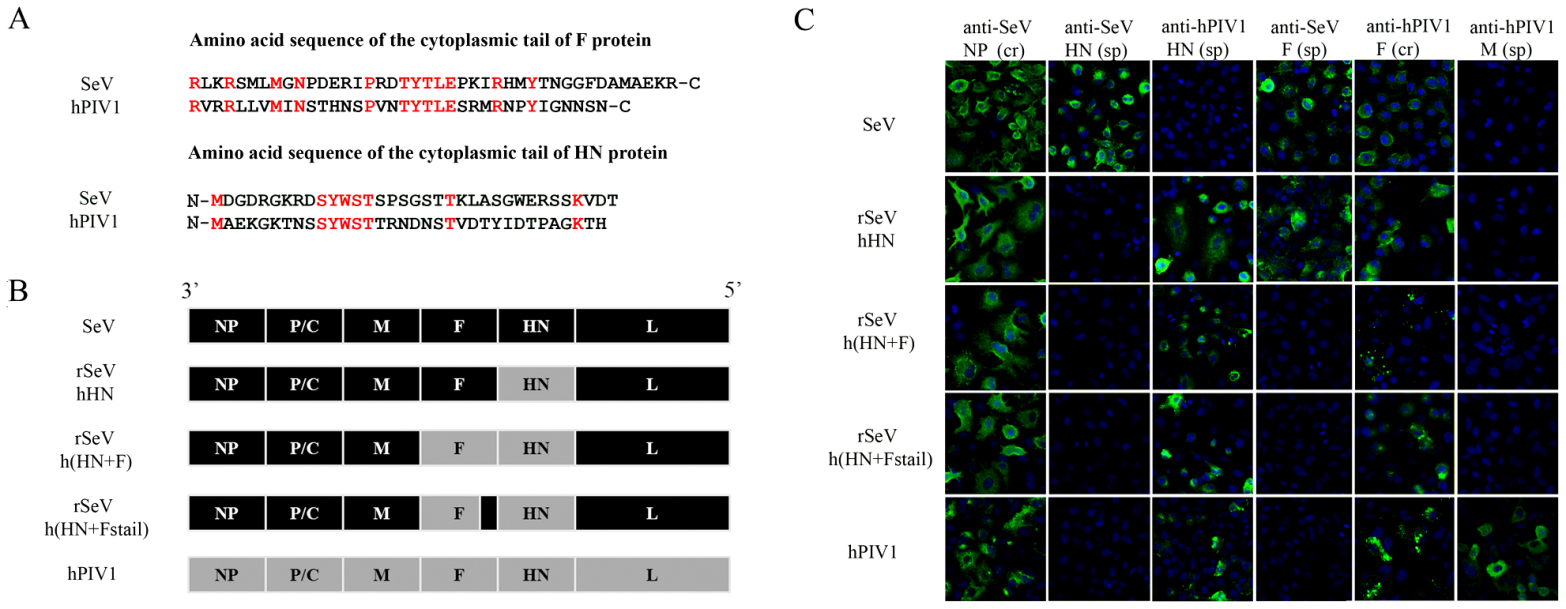
Rescue and characterization of rSeVhHN, rSeVh(HN+F) and rSeVh(HN+Fstail). (A) Aligned amino acid sequences of the F and HN cytoplasmic tails of hPIV1 and SeV. Conserved amino acids are highlighted in red. (B) Schematic diagram of rSeV genomes compared with wt SeV and hPIV1. With rSeVhHN, the SeV HN gene was replaced with that of hPIV1. In rSeVh(HN+F), both HN and F genes of SeV were replaced with those of hPIV1. For rSeVh(HN+Fstail), the entire cytoplasmic tail portion of hPIV1 F shown in Figure 1A was replaced with that of SeV F. (C) Expression of HN and F from rSeVs. IF analysis of A549 cells infected with wt SeV, hPIV1 or rSeV. Origins of HN or F were confirmed using cross reactive (cr) or specific (sp) mAb for NP (cr M52), HN (SeV-sp S16, hPIV1-sp P24), F (SeV-sp M38, cr P38) or M (hPIV1-sp P3) [12], [36], [41], [42], [43].

SeV and hPIV1 are highly homologous, and the overall homology of the glycoproteins is high (68% for F and 72% for HN). However, the amino acids in the cytoplasmic domains of F and HN are not well conserved (28% for F and 23% for HN, Fig. 1A) [30], [31]. The HN cytoplasmic tails of SeV and hPIV1 contain the an identical SYWST sequence in the middle of the tail domain, which we previously showed to be required for specific incorporation of HN to SeV [32]. The cytoplasmic tail of SeV and hPIV1 F protein also shares the TYTLE sequence, which may play a similar role as HN for specific incorporation into virions [32]. To determine the role and requirement of cytoplasmic tail sequences in the glycoproteins for assembly and virion formation, we created chimeric SeVs which contain hPIV1 HN (rSeVhHN) or both HN and F (rSeVh(HN+F)), in place of the SeV HN, or HN and F genes, respectively. In addition, rSeVh(HN+Fstail), containing a chimeric F composed of external and transmembrane domains from hPIV1 and the cytoplasmic tail (shown in Fig. 1A) from SeV, was created (Fig. 1B). We did not attempt to rescue rSeV containing only replacement of hPIV1 F because our previous study indicated that SeV HN is unable to promote fusion induced by hPIV1 F [33]. The HN and F regions of the recombinant viruses were sequenced and the presence of the designed HN and F genes was confirmed (data not shown). The expression of the specific viral proteins was confirmed by immunofluorescent (IF) assay of the cells infected with the viruses using monoclonal antibodies specific or cross-reactive to hPIV1 or SeV HN and F proteins (Fig. 1C).

**Figure 2:**
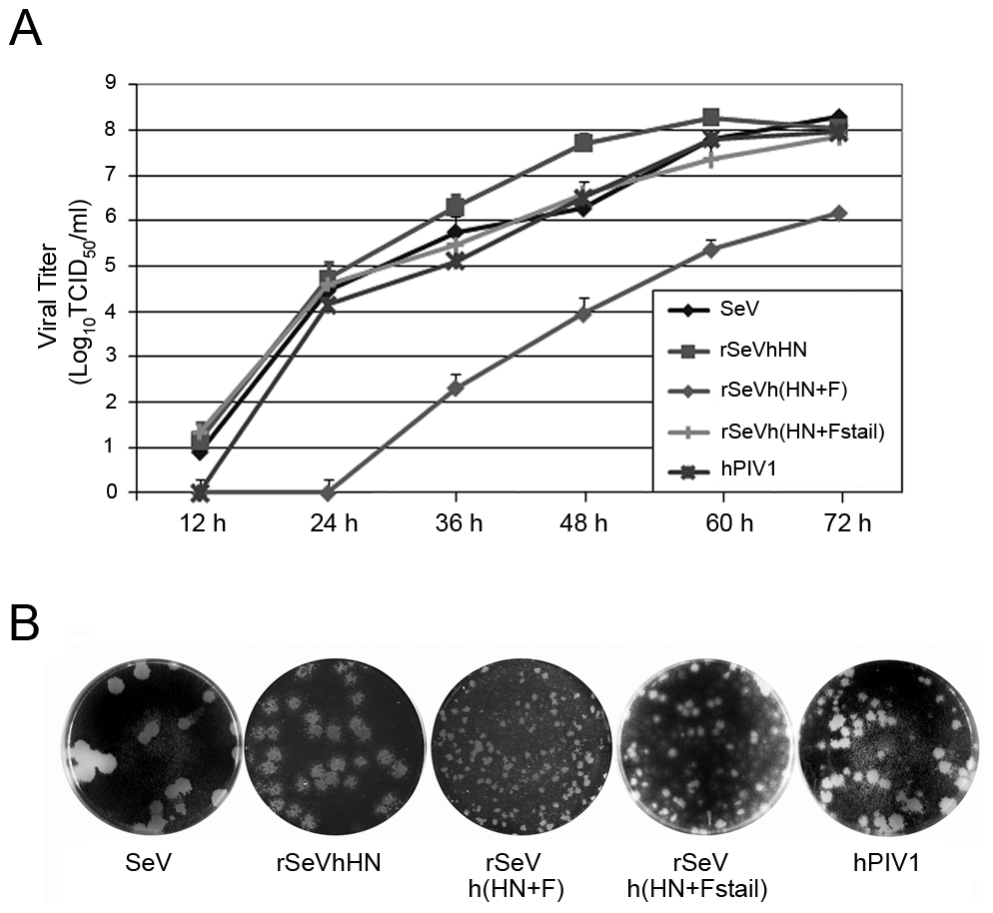
Virus growth kinetics and plaque formation of rSeVs. (A) Multi-step growth curve of the viruses in LLC-MK_2_ cells. Cells were infected with wt or chimeric viruses at MOI 0.01 and incubated at 34°C. Aliquots of infected cell supernatants were collected at indicated times after infection and viral titers of supernatants were determined in LLC-MK_2_ cells. (B) Plaque formation of the wt and rSeVs. LLC-MK_2_ cells were infected with SeV, rSeVhHN, rSeVh(HN+F), rSeVh(HN+Fstail) or hPIV1 and cultured at 34°C with medium containing agarose. Plaques were identified using crystal violet staining.

Next, we compared the growth kinetics of the chimeric viruses to wt SeV and hPIV1 in LLC-MK_2_ cells. Cells infected at an MOI of 0.01 were cultured in the presence of trypsin for 72 h at 34°C, which is the optimum temperature for hPIV1 growth due to HN heat stability [34]. Infectious virus at each time point was quantitated. The rSeVhHN and rSeVh(HN+Fstail) grew to similar titers as wt SeV and hPIV1, but the rSeVh(HN+F) showed delayed growth and an overall decrease in infectious virus yield (Fig. 2A). Consistent with the virus growth curve, the rSeVh(HN+F) exhibited a small plaque phenotype compared with rSeVhHN in a plaque assay (Fig. 2B). Plaque size of rSeVh(HN+Fstail) was apparently larger than rSeVh(HN+F), although it is still smaller than rSeVhHN. These data suggest that the difference in the HN cytoplasmic tails between SeV and hPIV1 has little effect on SeV production. In contrast, F cytoplasmic tail plays an important role in SeV growth and spread.

**Figure 3:**
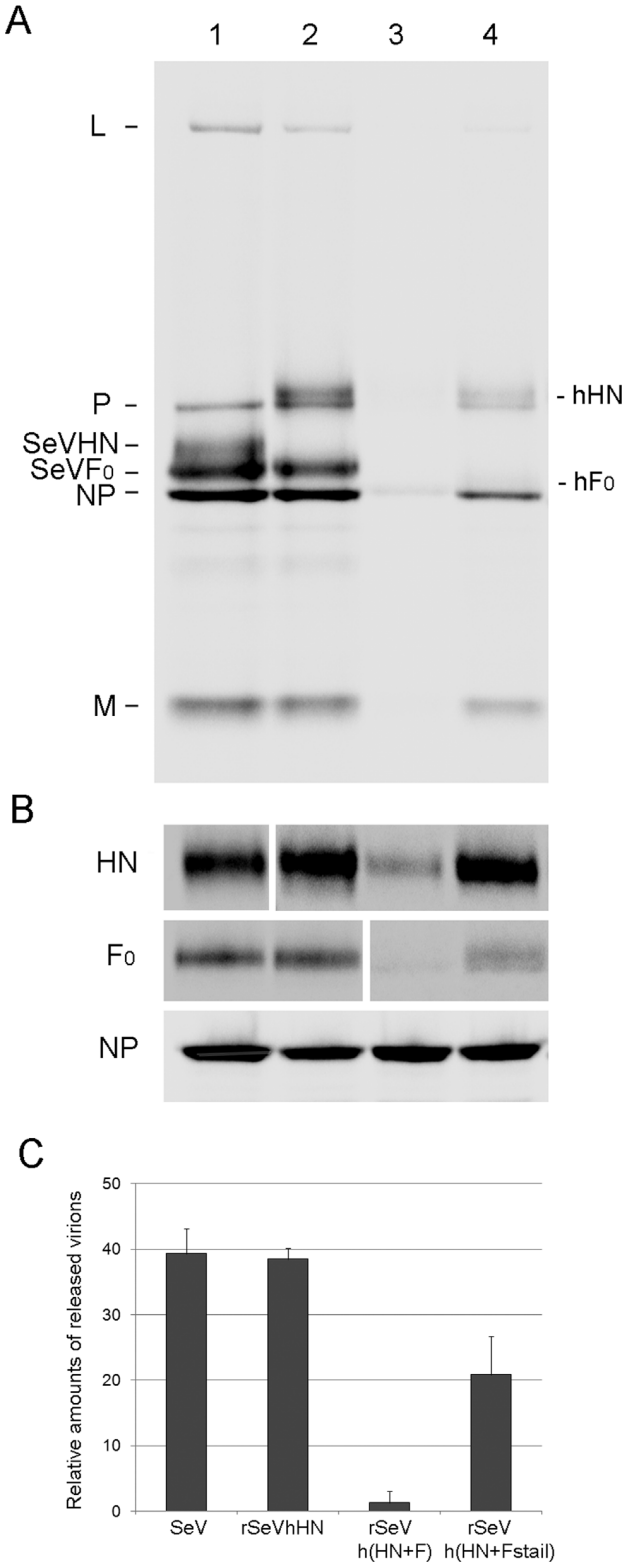
Virus production from infected cells. (A) Virion production from LLC-MK_2_ cells infected with SeV (lane 1), rSeVhHN (lane 2), rSeVh(HN+F) (lane 3), or rSeVh(HN+Fstail) (lane 4). Cells were infected at a MOI of 1 and labeled with [^35^S] Met/Cys for 16 h. Labeled progeny virions released from the cells were purified and analyzed by SDS-PAGE. (B) Viral proteins produced in infected cells. HN, F and NP proteins in [^35^S]-labeled cell lysates as described in (A) were immunoprecipitated using anti-SeV or hPIV1 HN, F or NP antibodies. (C) Amounts of NP in released virions (A) were quantified and shown as the average of three independent experiments with standard deviations.

To further characterize viral replication and production from cells, we determined the amounts of virions produced from infected cells, as well as viral protein levels in infected cells (Fig. 3). As with the virus growth curve and plaque phenotypes, virion production in cells infected with rSeVh(HN+F) (Fig. 3A, lane 3) was significantly lower than wt SeV (lane 1) or rSeVhHN (lane 2). The result was confirmed with three independent assays (Fig. 3C). rSeVh(HN+Fstail) which contains the cytoplasmic tail of SeV F was produced in a greater quantity than rSeVh(HN+F), highlighting the critical role of the F cytoplasmic tail in virus assembly and release. Cells infected with rSeVhHN, rSeVh(HN+F) and rSeVh(HN+Fstail) produced similar levels of NP to wt SeV, confirming the same level of viral infection. However, in contrast to NP, HN and F levels in rSeVh(HN+F)-infected cell lysates was significantly less than rSeVhHN. Expression of hPIV1 F from rSeVh(HN+F) is expected to be less than that of SeV F because of the long non-coding region of the hPIV1 F gene [35]. Level of hPIV1 F in cell lysate was significantly higher in rSeVh(HN+Fstail)-infected cells (Fig. 3B, lanes 3 and 4). Similarly, more hPIV1 HN was detected in rSeVh(HN+Fstail)-infected cells than in that of rSeVh(HN+F)-infected cells. Because rSeVh(HN+F) and rSeVh(HN+Fstail) differ only in the sequence of the F cytoplasmic tail, the result indicates a major contribution of the F cytoplasmic tail sequence for accumulation of both envelope glycoproteins. Our data also suggest that stable expression/accumulation of the envelope glycoproteins is required for efficient virion production from infected cells.

### Limited plasma membrane localization of HN and F in rSeVh(HN+F)-infected cells

**Figure 4:**
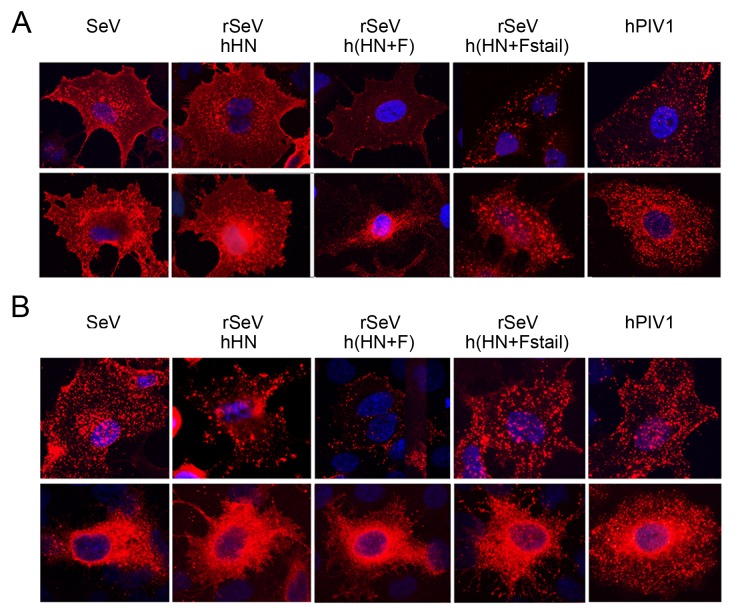
Surface and subcellular localization of HN and F proteins. A549 cells were infected with SeV, hPIV1 or rSeVs and incubated for 16 h at 34°C. Cells were then fixed and treated with mAbs against SeV or hPIV1 F (A) or HN (B) without (upper panels) or with (lower panels) permeabilization. Anti-mouse IgG-Texas Red was used as secondary.

We next visualized the localization of HN or F in the cytoplasm and at the plasma membrane in infected cells by IF. A549 cells were infected with the recombinant viruses and either permeabilized to observe intracellular protein localization or non-permeabilized to detect cell surface localization. The cell surface staining of F in rSeVh(HN+F)-infected cells showed very limited F at the plasma membrane compared with the F punctae staining visualized in hPIV1- or rSeVh(HN+Fstail)-infected cells (Fig. 4A). This shows that, in rSeVh(HN+F)-infected cells, F did not accumulate at the cell surface. Interestingly, the results for HN protein localization resembled that of F (Fig. 4B), suggesting that the HN in rSeVh(HN+F)-infected cells is unable to accumulate stably at the cell surface.

### Limited plasma membrane localization of M and NP proteins in rSeVh(HN+F)-infected cells

**Figure 5:**
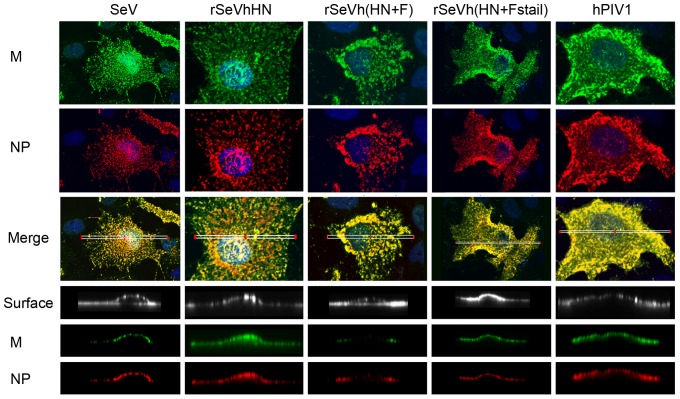
M and NP localization below the plasma membrane of infected cells. A549 cells were infected with the indicated viruses for 16 h at 34°C. Cells were processed for IF using anti-SeV NP mAb or M rabbit serum. Top 3 panels represent localization of M and NP. Merged images are deconvoluted z-stack images of the xy-plane created using an Olympus FV1000 confocal microcope. Bottom 3 panels show plasma membrane, M and NP and are z-stack reconstructions of the ROIs indicated in the whole cell images by white rectangles in the merged images.

Progeny virions are formed at the plasma membrane budding sites, and specific interactions between structural components, vRNP, M and envelope glycoproteins are considered to be essential. Since surface glycoproteins were limited in cells infected with rSeVh(HN+F), we next determined whether M and vRNP were accumulated below the plasma membrane in rSeVh(HN+F)-infected cells. A549 were infected with wt or recombinant viruses, the cell surface was biotinylated and processed for IF analysis using anti-SeV NP (Texas Red) or SeV M (FITC) Ab and streptavidin conjugated to Cy5 for cell surface staining. Using a confocal microscope, 3D reconstructions from deconvoluted z-stack images of the xy plane were analyzed and reconstructed to allow a lateral view of the cell (Fig. 5). In cells infected with wt SeV or hPIV1, accumulation of both M and NP was clearly detected. Similarly, rSeVhHN showed similar patterns of M and NP localization, suggesting that the difference in HN sequence between SeV and hPIV1 does not have a significant impact on the accumulation of M and NP in infected cells. As seen with the HN and F surface staining in rSeVh(HN+F)-infected cells, the localizations of NP and M below the plasma membrane were limited, signifying that the viral protein interactions necessary for assembly and virion formation are suboptimal in rSeVh(HN+F)-infected cells. Additionally, the NP and M in rSeVh(HN+F)-infected cells formed large aggregates within the cytoplasm, which differs from the more evenly distributed pattern observed with the other recombinant viruses. These results suggest that lack of envelope glycoproteins at the plasma membrane also affects the localization of vRNP and M.

### Lack of envelope protein accumulation does not affect vRNP trafficking in the cytoplasm

**Figure 6:**
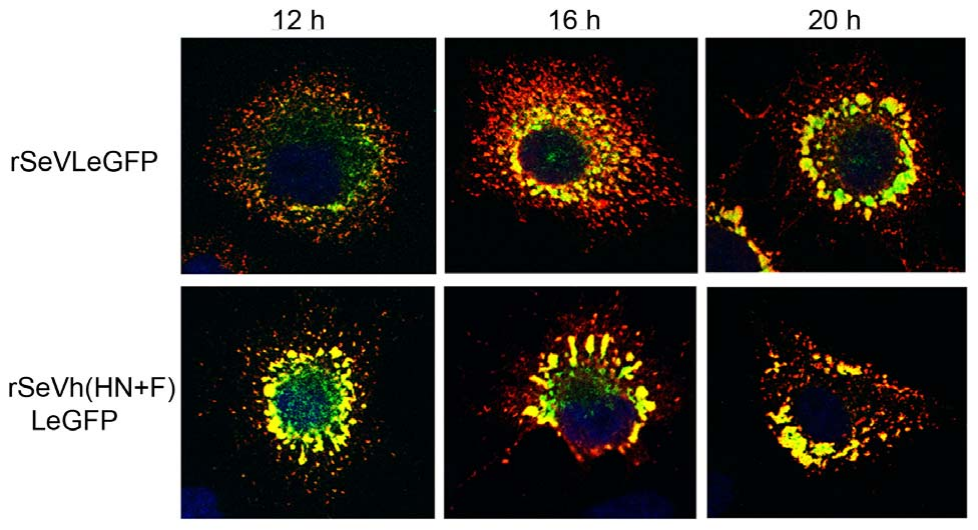
Accumulation of vRNP in rSeVh(HN+F)LeGFP-infected cells. A549 infected with rSeVLeGFP or rSeVh(HN+F)LeGFP were fixed at the indicated hours after infection, and processed for IF to detect M protein (red).

In our previous study, we rescued a rSeV with eGFP fused to the L protein (rSeVLeGFP), and used live cell digital video microscopy to visualize movement of vRNP [6]. Using this virus, we showed that vRNP transport is microtubule dependent and involves the recycling endosome pathway. To determine if the reduced vRNP accumulation at the plasma membrane and formation of large vRNP aggregates in the cytoplasm of rSeVh(HN+F)-infected cells were due to inefficient vRNP transport, we rescued a rSeVh(HN+F)LeGFP, which expresses L fused to eGFP in the background of rSeVh(HN+F). The accumulation of vRNP-M complexes and vRNP movement were compared between rSeVLeGFP and rSeVh(HN+F)LeGFP at different times after infection. At 12 h p.i., vRNP-M complexes had already formed in the cytoplasm of the rSeVh(HN+F)LeGFP-infected cells, unlike rSeVLeGFP nucleocapsid, which exhibited a primarily punctate appearance with some larger areas of accumulated vRNP (Fig. 6). In spite of the large vRNP accumulations, small punctae were also present in the rSeVh(HN+F)LeGFP-infected cells and the movement of these nucleocapsids was similar to that of rSeVLeGFP (Videos S1 and S2). The average velocities of LeGFPs which traveled greater than 2 µm in length in rSeVh(HN+F)LeGFP-infected cells were 0.24–0.73 µm/sec, which is similar to the kinetics observed for rSeVLeGFP [6]. The movement of these vRNPs was indicative of movement along microtubules, similar to that of rSeVLeGFP. These results suggest that trafficking of vRNPs in the cytoplasm of rSeVh(HN+F)LeGFP-infected cells is unaffected, however, the vRNPs are recycled back into the cytoplasm due to a deficiency in the accumulation of M and envelope glycoproteins at the plasma membrane.

## Discussion

In this study, we determined the role of glycoproteins in virus assembly and formation in the context of an infection by taking advantage of the low amino acid homology of HN and F cytoplasmic tail sequences between SeV and hPIV1, which share only 23 and 28% identity, respectively. Our data indicate that 1) the difference in the HN cytoplasmic tail sequence does not affect SeV growth and virion formation, 2) the F cytoplasmic tail sequence plays a major role in accumulation of both HN and F at the cell surface, and 3) lack of a specific F cytoplasmic sequence results in a reduction in the accumulation of M and vRNP at the plasma membrane and overall virion production. These findings highlight the critical role of F cytoplasmic tail in virus assembly and suggest that specific interactions between F cytoplasmic tail and M are a key factor for recruiting all viral structural components to plasma membrane budding sites.

The important role of SeV F in virus budding is consistent with our previous study showing that expression of SeV F, but not HN by itself induced VLP formation [36]. The major contribution of F, but not HN in SeV virion production was also suggested in a previous study using siRNA to knock down HN or F protein expression [24]. Comparison of virion production from cells infected with rSeVh(HN+F) and rSeVh(HN+Fstail) clearly indicates that the SeV F cytoplasmic tail includes a critical domain necessary for the formation of progeny virions (Fig. 2). Replacement of the hPIV1 F cytoplasmic tail with that of SeV significantly increased HN and F cell surface accumulation (Fig. 4) and virion production (Fig. 3), suggesting that a specific interaction between F cytoplasmic tail and M plays a major role in virus assembly and budding site formation. Although SeV and hPIV1 F share the TYTLE sequence in the cytoplasmic tail (Fig. 1A), other parts of the sequence are likely to be responsible for the lack of hPIV1 F accumulation at the surface of rSeVh(HN+F)-infected cells. F proteins located at the cell surface could be internalized through lack of a specific interaction with M, and due to the specific HN-F interaction, HN may also be internalized in rSeVh(HN+F)-infected cells resulting in reduced accumulation of both HN and F proteins at the cell surface. Alternatively, it is also possible that efficient translocation of F to the plasma membrane through the exocytic pathway may require a stable interaction with a specific viral M protein.

In sharp contrast, rSeVhHN which expresses hPIV1 HN and other components of SeV, replicated and assembled virions as efficiently as wt SeV. SeV and hPIV1 HN cytoplasmic tails share no identity except the SYWST sequence (Fig. 1A), which we previously identified to be required for specific incorporation of HN proteins into progeny SeV [32]. It is possible that the common SYWST sequence is responsible for the interaction with both SeV and hPIV1 M proteins. Our previous data support this possibility since chimeric Newcastle disease virus HN containing the cytoplasmic SYWST sequence was incorporated into progeny SeV virions [32]. The hPIV1 HN and SeV F are considered to function coordinately for infection, because expression of hPIV1 HN and SeV F from cDNAs induced membrane fusion [33]. However, in rSeVh(HN+F)-infected cells, reduction in both HN and F surface expression suggests that SeV M-hPIV1 HN interaction may not be sufficient to retain hPIV1 HN at the surface. In other words, HN may be able to accumulate at the surface through interactions with F, which can be accumulated at the surface through the interaction of its cytoplasmic tail and M. In this case, a specific F cytoplasmic tail and M interaction is the major factor for accumulation of envelope proteins at the budding site, which is likely to be required for efficient budding and virion production.

Although our data and previous studies support a major role for F in virus assembly and budding, contribution of the envelope glycoprotein cytoplasmic tails in virus budding seems to differ between paramyxoviruses. In the case of parainfluenza virus 5 (PIV5), truncation of F cytoplasmic tail affects budding less than that of truncations in HN cytoplasmic tail in reducing viral budding and production [17], [19]. IF study of the cells infected with HN cytoplasmic tail-truncated viruses showed HN and F distributed all across the cell surface, unlike wt PIV5-infected cells where HN and F formed highly localized patches on the cell surface, suggesting that reduced budding could be due to redistribution of the membrane glycoproteins at the surface [17].

It is well established that viral M protein is the key driver of virus assembly and budding. M protein links together the major structural components of the virus through interaction with vRNP and envelope glycoproteins via their cytoplasmic tails. In rSeVh(HN+F)-infected cells, in addition to HN and F, M and vRNP accumulation at the plasma membrane was reduced (Fig. 5). IF analysis of cells infected with rSeVh(HN+F) showed extensive localization of M with vRNP in the cytoplasm as large aggregates. This lack of accumulation of vRNP at the plasma membrane is unlikely due to a defect in vRNP trafficking to the plasma membrane, because movement of vRNP in rSeVh(HN+F)LeGFP-infected cells seems to be as efficient as rSeVLeGFP (Videos S1 and S2). M and vRNP showed extensive co-localization at the plasma membrane of cells infected with SeV, hPIV1, rSeVhHN, and rSeVh(HN+Fstail), but not with rSeVh(HN+F) (Fig. 5). M and vRNP co-localize mainly in large clusters in the cytoplasm in rSeVh(HN+F)-infected cells. Therefore, vRNP accumulation at assembly sites may require a stable interaction with M at the plasma membrane, which could be provided by specific interaction of M with the F cytoplasmic tail. It is not known if F cytoplasmic tail directly interacts with vRNP at the plasma membrane. A recent cryoelectron tomography study of Newcastle disease virus (NDV) showed that the glycoproteins were anchored in the gaps between repeating units in the matrix array [9]. The helical vRNP were also detected in association with the matrix arrays, which is consistent with the idea that M protein is a key organizer of virus assembly. Although vRNP association to the glycoprotein tails in the membrane lacking matrix arrays was not described in this study, it is also possible that F cytoplasmic domain contributes to vRNP association to the matrix array.

Overall, characterization of the recombinant SeVs containing envelope glycoproteins from hPIV1 highlights the importance of the F cytoplasmic tail in recruitment/accumulation of viral structural components at assembly sites on the infected cell membrane. Clearly, our study indicates that the F cytoplasmic domain critical for virion production is not shared between SeV and hPIV1. It is predicted that lack of a specific interaction between the F cytoplasmic tail with M causes inefficient recruitment and enrichment of structural components at budding sites, which is essential for virion assembly and production.

## Materials and Methods

### Cell and viruses

LLC-MK_2_, A549, HeLa and HeLa T4^+^ [37] cells were cultured in Dulbecco's modified Eagle's medium (DMEM) with 8% fetal calf serum (FCS). SeV (strain Enders), hPIV1 (strain C-35) and the recombinant SeVs: rSeVhHN, rSeVh(HN+F), rSeVh(HN+Fstail), rSeVLeGFP [6], and rSeVh(HN+F)LeGFP were grown in LLC-MK_2_ cells in DMEM supplemented with acetylated trypsin (2 μg/ml). Recombinant vaccinia virus vTF7.3, which expresses T7 polymerase [38] was grown in HeLa cells.

### cDNA synthesis and cloning

The full genome cDNA of rSeV (pSeV(E), strain Enders) [39] was mutated at the non-coding regions between M and F, F and HN, and HN and L to include unique restriction sites for *FseI*, *NotI*, and *AscI*, respectively. The SeV HN and F genes in pSeV(E) were replaced with those of hPIV1 HN and F cDNAs produced by PCR using primers containing appropriate restriction sites. The SeV cDNAs containing the hPIV1 HN gene, and both hPIV1 HN and F, were designated as pSeVhHN and pSeVh(HN+F). The hPIV1 F cDNA encoding the SeV cytoplasmic tail sequence was constructed using PCR for gene splicing by overlap extension [40]. pSeVh(HN+Fstail) was produced by replacing the hPIV1 F gene in pSeVh(HN+F) with that of the chimeric F cDNA. The pSeVh(HN+F)LeGFP was constructed by replacing the *XhoI/KpnI* fragment of pSeVh(HN+F) with that of pSeVLeGFP, which included the L gene tagged with eGFP [6].

### Rescue of rSeVhHN, rSeVh(HN+F), rSeVh(HN+Fstail), and rSeVh(HN+F)LeGFP

The recombinant SeVs were rescued as described previously [35]. Briefly, HeLa T4^+^ cells infected with vTF7.3 were transfected with 2 μg of pSeVhHN, pSeVh(HN+F), pSeVh(HN+Fstail), or pSeVh(HN+F)LeGFP together with supporting plasmids pTF1SeVNP (1μg), pTF1SeVP (1 μg), and pTF1SeVL (0.1 μg) by Lipofectamine 2000 (Invitrogen). After 36 h incubation in DMEM supplemented with 0.15% bovine serum albumin plus araC (40 µg/ml), the cells were treated with trypsin and overlayed onto LLC-MK_2_ cells and cultured in DMEM containing trypsin. The rescued viruses were plaque purified in LLC-MK_2_ cells, and the stock viruses were grown in LLC-MK_2_ cells.

### Virus growth in LLC-MK_2_ cells

Cells in a six-well plate were infected with the recombinant SeVs at an MOI of 0.01 and incubated at 34°C in 2 ml DMEM containing 0.15% BSA and acetylated trypsin (2 µg/ml). Culture medium (200 µl) was harvested every 12 h after infection. Virus titers in the supernatant were determined by tissue culture infection in LLC-MK_2_.

### Plaque assay

LLC-MK_2_ cells were infected with the viruses and cultured at 34°C with medium containing acetylated trypsin (2 µg/ml) and 0.9% SeaKem LE agarose (Lonza). Plaques were identified using crystal violet staining.

### Purification of produced virions and radioimmunoprecipitation (RIP)

LLC-MK_2_ cells infected with the viruses at MOI 1 for 16 h were labeled with [^35^S] Met/Cys (Perkin Elmer) for an additional 16 h. The supernatants were collected and purified by ultracentrifugation over 40% glycerol cushions at 190,000× *g* for 2 h at 4°C. The virus pellet was resuspended in Laemmli sample loading buffer and analyzed by SDS-PAGE. For immunoprecipitation of viral proteins, labeled cells were lysed with TNE buffer (10 mM Tris [pH 7.4], 150 mM NaCl, 0.5% NP-40, and 1 mM EDTA) and incubated with Protein G-Dynabeads pre-incubated with anti-SeV NP (M52), HN (M2) or F (M38) or hPIV1 NP (P19), HN (P24) or F (P12) monoclonal antibody (mAb). The samples were analyzed by SDS-PAGE. Band intensities were quantified with BioRad Quantity One software and the amount of NP detected in purified virions was normalized to NP expressed in cell lysates.

### Immunofluorescence (IF) assays and confocal microscopy

Localization of viral proteins in cells infected with wt and recombinant SeVs was determined by IF assay using confocal microscope. A549 cells were infected with SeV, rSeVhHN, rSeVh(HN+F), rSeVh(HN+Fstail) or hPIV1 at MOI 0.8. After 16 h, cells were fixed with 4% paraformaldehyde (PFA) for 15 min and either permeabilized with 0.1% Triton X-100 for 10 min at room temperature (RT) or left unpermeabilized. HN and F proteins were detected by reaction with α-hPIV1 HN (P24), α-SeV HN (M2), α-hPIV1 F (P12) and α-SeV F (M38) followed by goat anti-mouse Texas Red (Molecular Probes) 41,42,43. For detection of M and NP, permeabilized cells were reacted with anti-M rabbit serum followed by anti-rabbit IgG-FITC, and anti-NP mAb followed by anti-mouse Texas Red. Z-stack reconstructions of the samples were obtained using an Olympus FV1000 confocal microscope with a 63× oil immersion objective. These experiments were repeated at least three times and representative images are shown in the Figures. Parameters for each experiment, such as exposure length and laser power, were kept the same by re-loading the specific parameters from previous experimental files.

### Live cell trafficking of vRNP

HeLa cells in a ΔT35 dish (Bioptechs) were infected with rSeVLeGFP or rSeVh(HN+F)LeGFP for 18 h, then the movement of LeGFP was recorded using a Leica DMIRB inverted fluorescence microscope equipped with Image-Pro Plus software (Mediacybernetics) while maintaining the cells at 37°C on a ΔTC3 temperature-controlled stage. The video image data were analyzed using NIH ImageJ software.

## Supporting Information

Video S1 **Movement of LeGFP in rSeVLeGFP-infected A549 cells.** Live infected cells were observed using a Leica DMIRB inverted fluorescence microscope equipped with Image-Pro Plus software. The movements of LeGFP were recorded while maintaining the cells at 37°C.
(AVI)

Video S2 **Movement of LeGFP in rSeVh(HN+F)LeGFP-infected A549 cells.** Live infected cells were observed using a Leica DMIRB inverted fluorescence microscope equipped with Image-Pro Plus software. The movements of LeGFP were recorded while maintaining the cells at 37°C.
(AVI)
